# Cost-effectiveness of a multifaceted implementation strategy for the Dutch multidisciplinary guideline for nonspecific low back pain: design of a stepped-wedge cluster randomised controlled trial

**DOI:** 10.1186/s12889-015-1876-1

**Published:** 2015-05-31

**Authors:** Arnela Suman, Frederieke G. Schaafsma, Petra J.M. Elders, Maurits W. van Tulder, Johannes R. Anema

**Affiliations:** Department of Public and Occupational Health, VU University medical centre, EMGO+ Institute for Health and Care Research, PO Box 7057, 1007 MB Amsterdam, The Netherlands; Research Centre for Insurance Medicine, Collaboration between AMC-UMCG-UWV-VUmc, Amsterdam, The Netherlands; Department of General Practice and Elderly Care Medicine, VU University medical centre, EMGO+ Institute for Health and Care Research, Amsterdam, The Netherlands; Department of Health Sciences, Section Health Economics and Health Technology Assessment, VU University, Amsterdam, The Netherlands

**Keywords:** Low back pain, Back Beliefs, Guideline implementation, Multifaceted implementation strategy, Stepped-wedge trial, RCT, Cluster randomisation, Economic evaluation, Work Disability, Work Status

## Abstract

**Background:**

Low back pain (LBP) is one of the most prevalent and expensive health care problems in industrialised countries. LBP leads to high health care utility and productivity losses; leaving the individual, the employer, and society with substantial costs. To improve the care for LBP patients and reduce the high societal and financial burden of LBP, in 2010 the ‘Multidisciplinary care guideline for nonspecific low back pain’ was developed in the Netherlands. The current paper describes the design of a study aiming to evaluate the (cost-) effectiveness of a multifaceted strategy to implement this guideline.

**Methods:**

In a cluster-randomised controlled trial, the (cost-) effectiveness of a multifaceted implementation strategy will be compared to passive guideline dissemination. Using a stepped-wedge approach, participating general practitioners, physiotherapists, and occupational physicians are allocated into clusters and will attend a multidisciplinary continuing medical education training session. The timing these clusters receive the training is the unit of randomisation. LBP patients visiting the participating health care providers are invited to participate in the trial and will receive access to a multimedia intervention aimed at improving beliefs, cognitions, and self-management. The primary outcome measure of this study is patient back beliefs. Secondary outcome measures on patient level include pain, functional status, quality of life, health care utility, and productivity losses. Outcome measures on professional level include knowledge and attitude towards the guideline, and guideline adherence. A process evaluation for the implementation strategy will be performed among the health care providers and the patients. Furthermore, a qualitative subgroup analysis among patients with various ethnic backgrounds will be performed.

**Discussion:**

This study will give insight into the (cost-) effectiveness of a multifaceted implementation strategy for the Dutch multidisciplinary guideline for non-specific back pain to improve outcomes on patient and professional level. The valuable information gained with this study may prove useful for policy-makers, health care providers, and researchers who are in the process of reducing the burden of back pain on individuals and society.

**Trial Registration:**

Netherlands Trial Register (NTR): NTR4329. Registered December 20th, 2013.

**Electronic supplementary material:**

The online version of this article (doi:10.1186/s12889-015-1876-1) contains supplementary material, which is available to authorized users.

## Background

Low back pain (LBP), with a lifetime prevalence of more than 70 % in industrialized countries, is one of the most prevalent and expensive health care problems worldwide [1]. It is estimated to be responsible for 83 million years lived in disability, making it globally the leading cause of disability [1–3]. The global age-standardized point prevalence (from 0 to 100 years of age) for LBP was estimated to be 9.4 % in 2010, while it was, with a 15 % prevalence rate, highest in Western Europe [3]. Although LBP is highly prevalent, it is a relatively innocent health problem. In 85 % of people the pain is not attributable to pathology or neurological damage, and LBP is therefore considered to be self-limiting (recovery rate 90 % within 6 weeks) [1]. Thus, for the majority of patients, diagnostic imaging, and referrals to specialist medical care are not indicated. On the contrary, studies have shown that referral of these patients for diagnostic imaging or consultation with a medical specialist may lead to chronicity, disability, and medicalization [4–6].

Nevertheless, health care utility and referral rates due to LBP remain high. In 2011, over 5 million visits to general practitioners (GPs) and over 1 million visits to physiotherapists (PTs) were reported in the Netherlands [7]; where LBPs annual economic burden amounts to €3.5 billion, of which 12 % is due to direct health care costs (i.e., health care utility, diagnostics, drugs, and treatment procedures) and 88 % due to productivity losses, absenteeism and disability [7]. In 2007, 6 million sick leave days (7 % of total annual sickness reports), and 100 k work disability benefit claims were registered in the Netherlands [7, 8]. LBP remains a significant financial as well as a societal burden for other industrialized countries as well. Annual costs due to LBP have been reported to vary between AU$9.17 billion in Australia [9] to £12.3 billion in the UK [10], and as much as US$91 billion in the United States [11]. The vast majority of these costs are related to disability and productivity losses [12].

Despite various efforts to discourage and reduce (early) direct health care utility for LBP, there has been no decline in the high number of ineffective and costly referrals. Various underlying causes for the failure of these previous efforts to reduce LBP and its effects on society can be identified. Research has shown that a considerable proportion of LBP patients predominantly focus on pain and other physical complaints, and that they are less attentive to restoration of functional capacities [13]. Patients’ attitudes towards back pain and knowledge of their illness are important determinants of this problem. In particular, cognitions such as fear avoidance beliefs (i.e., patients avoid physical movement out of fear of pain) and passive coping strategies play an important role in disabling LBP in many patients [8, 14, 15]. The high rates of referrals, disability claims, and health care utility underline this evident focus on pain, instead of functional restoration of LBP patients.

Therefore, further measures need to be taken to reduce the burden of LBP, and studies have shown that multidisciplinary approaches are (cost-) effective in reducing pain, disability, and fear avoidance beliefs, and that they improve work status, functional recovery and quality of life of patients with LBP [16, 17]. These improvements may reduce referrals for diagnostic imaging, outpatient medical specialist consultations, or surgeries for LBP. Unfortunately, initiatives to enhance multidisciplinary care, and to reduce these costly and ineffective referrals for LBP have not proven to be successful yet [5]. Patients have reported poor communication and collaboration between health care providers (HCPs) to be an important barrier to recovery, as it leads to conflicting treatment advices and poor coordination of care [6, 18].

To improve care for LBP patients and reduce the high societal and financial burden of LBP, in 2010 the ‘Multidisciplinary care guideline for nonspecific low back pain’ was developed in the Netherlands [6]. This guideline recommends multidisciplinary collaboration and communication between HCPs and emphasizes the importance of physical activity and return-to-work of LBP patients. The aim of the current study is to evaluate the implementation of this guideline in the daily practice of GPs, PTs, and occupational physicians (OPs) in the Amsterdam region, the Netherlands. This paper describes the design of a stepped-wedge cluster randomised controlled trial with an economic evaluation alongside to evaluate the (cost-) effectiveness of a patient and professional based multifaceted implementation strategy for this guideline.

## Methods

The methods of this study are described according to the CONSORT statement for cluster-randomised trials [19].

### Study design

**Fig. 1:**
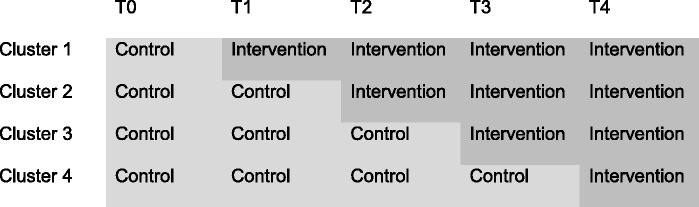
Design of the stepped-wedge trial

This study is a stepped-wedge cluster-randomised controlled trial. A multifaceted implementation strategy for the Dutch multidisciplinary care guideline for nonspecific LBP will be compared to passive dissemination of the guideline. The implementation strategy will be targeted at both health care professionals (i.e., GPs, OPs and PTs) and patients. The HCPs are allocated to one of four clusters, which are compiled based on the HCPs’ geographic proximity to each other. This grouping allows for minimisation of contamination between the participating HCPs. A stepped-wedge design is applied to assure stepwise implementation of the guideline in all of the participating HCPs (Fig. 1). Patients are allocated according to their GP/PT allocation, i.e., patients registered within a practice that is in the control group at time of enrolment will automatically be allocated into the control group for patients.

The Medical Ethics Committee of the VU University medical centre assessed this study design and procedures, and in accordance with the local regulatory guidelines and standards for human subjects protection in the Netherlands (Medical Research Involving Human Subjects Act [WMO], 2005), this study proved to be exempt from further medical ethical review.

### Participants

#### Health care providers

This study comprises 25 GP practices (accounting for 53 individual GPs), 19 PT practices (accounting for 43 individual PTs), and 37 OPs. Inclusion criteria for health care providers are: practising within the municipality of Amsterdam, and regularly working with patients with LBP. Participating HCPs are allocated to one of four clusters based on defined geographical areas, which is the eligibility criterion for clusters in this trial.

#### Patients

Five hundred patients with LBP will be included to participate in this study. All patients registered in the practices of participating GPs and PTs receive an information leaflet about this study, and a recruitment letter from their GP/PT. Furthermore; PTs have information and recruitment posters and leaflets in their practices. Patients interested in participation can apply directly via e-mail or using a postal reply card at the research assistants’ office, where eligibility criteria are assessed prior to inclusion.

Patients are eligible if they have a minimum age of 18 and a maximum age of 75 years, have access to the Internet, have visited their GP or PT due to back complaints no later than 3 months prior to inclusion, and are diagnosed with nonspecific LBP. Nonspecific LBP is defined as low back pain (with or without motor and/or sensory deficits in one or both legs) that is not caused by underlying specific pathology (red flags), i.e., a tumour, (osteoporotic) vertebral fracture, ankylosing spondylitis, and cauda equina syndrome.

Patients having the following characteristics will be excluded from this study: serious long existing comorbidity, i.e., Alzheimer’s disease, Multiple Sclerosis, Parkinson’s disease, ALS, CVA (diagnosed 1 year prior to inclusion up to moment of inclusion), confirmed pregnancy (identified 1 year prior to inclusion up to moment of inclusion), malignancy (diagnosed 5 years prior to inclusion up to moment of inclusion), and severe psychiatric disorders, i.e., schizophrenia and bipolar disorder.

In accordance with the local regulatory guidelines and standards for human subjects protection in the Netherlands (Medical Research Involving Human Subjects Act [WMO], 2005), and the assessment of the Medical Ethics Committee of the VU University medical centre, no written informed consent for participation was necessary to be obtained from participants.

### Interventions

#### Passive dissemination of the guideline

In this study, a multifaceted implementation strategy (intervention group) targeted at both the patients and the professionals will be compared to passive dissemination of the guideline (control group). For HCPs, passive dissemination encompasses digital dissemination of the guideline (i.e., HCPs will receive an e-mail with this guideline in PDF format attached). Patients are offered a website on which solely the brief patient information from the guideline is published.

#### Multifaceted implementation strategy for health care providers

HCP clusters in the intervention arm will be invited to participate in a multidisciplinary continuing medical education (CME) training session that is developed in close collaboration with a professional educationalist. The training module meets the educational requirements of the Dutch physicians and physiotherapists associations.

The training module focuses on improving collaboration and communication between various HCPs, which is practiced by means of a so called barriers and facilitators carousel, in which several small groups of HCPs first discuss the barriers they encounter in collaborating and communicating with each other. The groups then interchange these barriers and discuss strategies to overcome them. In a plenary session, the HCPs then exchange their group findings and agree upon a set of practical strategies for dealing with the encountered barriers. Furthermore, communication skills needed for managing patients with nonspecific LBP are trained by means of a case study that dictates a role-play. The case study presents a complicated case of LBP in which yellow flags (i.e., psychosocial risk factors) and blue flags (i.e., occupational risk factors) play an important role. In this role-play, the HCPs swap professions with each other and play a role from the perspective of another HCP, allowing them to think outside their own frames of reference, while they also learn to identify important yellow and blue flags and act upon them in a multidisciplinary manner. During the role-play, the HCPs are encouraged to practice the previously agreed upon strategies. After the training module, HCPs repeatedly (directly after the training, and after 3, 6, and 12 months) receive a reminder about the strategies by e-mail, along with a social map of contact details of all HCPs that attended the specific training. The training is conducted one time per cluster and takes 2,5 h. It is organized and given by at least one member of the research team (in order to assure scientific quality), and one practicing HCP (to ensure relevance and connection to daily practice).

Finally, HCPs will gain access to an interactive website, containing information on LBP and LBP guidelines, updates on the study, and a HCP forum.

#### Multifaceted implementation strategy for patients

Patients randomised into the intervention arm will gain access to an informative website aimed at reducing patients’ negative back beliefs and improve their cognitions. The website provides comprehensive information about LBP, such as practical advices (e.g., on self-management), working and returning to work with LBP, exercise tips and possibilities to contact researchers, HCPs, and other patients by means of a forum and social media. An important part of the implementation strategy and this website are short video messages in which actors and HCPs share their fictional experience with LBP and provide tips on self-management of and working with LBP. These videos are based on the effective Australian mass media campaign ‘Back Pain: Don’t Take It Lying Down’ [20]. The website is also available in a mobile version to be visited on any electronic device, e.g., a smartphone or tablet.

### Objectives

The primary objective of this study is to evaluate the (cost-) effectiveness of the multifaceted, patient and professional based strategy for implementation of the Dutch multidisciplinary care guideline for nonspecific LBP on patients’ back pain beliefs and expectations. The secondary objective is the improvement of functional status and reduction of (work) disability of patients with LBP, and improvement of guideline adherence by HCPs.

### Outcomes

#### Outcomes on patient level

The primary outcome measure of this study is the back beliefs and expectations of patients with LBP, which will be assessed on individual level using the Back Beliefs Questionnaire [21]. Secondary outcome measures on patient level include functional status measured with the RDQ-24; quality of life measured with the EuroQol questionnaire; level of pain measured using an adapted form of the PCI questionnaire, and health care utility and productivity losses measured using the PRODISQ and TIC-P questionnaires. Measurements at patient level will take place at baseline and after 3, 6, and 12 months follow-up.

#### Outcomes on HCP level

Health care provider outcomes are measured at the level of the individual HCP. Using questionnaires, HCP knowledge of and attitudes toward the guideline will be measured, as well as level of perceived self-efficacy on multidisciplinary communication and collaboration. Measurements on HCP level will take place at baseline and after 3, 6 and 12 months follow-up.

Furthermore, guideline adherence among GPs will be assessed using performance indicators (Table 1). These professional behaviours are considered indicators for adherence to this guideline, because the guideline recommends a watchful waiting approach for acute LBP, and a multidisciplinary approach to treatment for chronic LBP, while it discourages (early) referrals of LBP patients for diagnostics and specialist medical care.

**Table 1:** Performance indicators to measure guideline adherence among GPs

Performance indicators for LBP	Operationalization
Referral to consultation with medical specialist (neurologist and/or orthopaedist)	Referrals as percentage of total consultations for non-specific LBP per GP, reported separately for both specialists
Referral for diagnostic imaging	Referrals for MRI, x-ray or CT as percentage of total consultations for non-specific LBP per GP, reported separately for every imaging technique
Inquiries about psychosocial risk factors (clinical yellow flags)	Consultations where psychosocial risk factors were discussed and reported, as percentage of total consultations for non-specific LBP per GP
Referral to psychological consultation as indicator for multidisciplinary collaboration	Referrals as percentage of total consultations for non-specific LBP per GP
Inquiries about work-related risk factors (occupational blue flags)	Consultations where occupational risk factors were reported as percentage of total consultations for non-specific LBP per GP
Referral to and/or contact with OP as indicator for multidisciplinary collaboration	Consultations where referral to and/or contact with OP was made as percentage of total consultations for non-specific LBP per GP, reported separately for referral to OP and contact between GP and OP

To enhance quality of the data, all data from questionnaires will be collected using online questionnaires, which are programmed to reduce impossible and missing values. Data on performance indicators will be gathered using software developed to select all GP reports on consultations with patients having LBP.

#### Process evaluation

The Linnan and Steckler framework for process evaluation of public health interventions and research will be used to evaluate the implementation process for this study at patient and HCP level [22]. The feasibility of the implementation strategy, barriers and facilitators for implementing the guideline, and the satisfaction of the participants (HCPs as well as patients) with the intervention are measured using questionnaires. In order to gain more in-depth knowledge on the satisfaction and experiences of the participants, semi-structured qualitative interviews with both HCPs and patients are conducted.

#### Subgroup analysis

Subsequent to the process evaluation, a subgroup evaluation analysis amongst participating patients will be performed. Using an explorative qualitative design with semi-structured interviews, 10 patients from four ethnic backgrounds (i.e., Dutch, Moroccan, Turkish, and Surinam, which are the most common ethnicities in the city of Amsterdam) will be interviewed to gain insight into their experiences with this study (e.g., the recruitment process, the intervention received). The differences in experiences between these ethnic groups will be mapped in order to gain insight into possible barriers and facilitators in involving various ethnic groups in future (implementation) research.

### Sample size

The sample size calculation is based on a hypothesized 10 % improvement of the primary outcome measure back beliefs of LBP patients (measured using the Back Beliefs Questionnaire). An intra-class correlation coefficient (ICC) of 0.05 is applied to adjust for the cluster randomisation design. Assuming a 10 % improvement of a mean score of 26.5 (95%BI 26.1-26.8, SD 6) on the Back Beliefs Questionnaire, and applying an ICC of 0.05, the necessary sample size amounts to 500 patients. This calculation takes into account a dropout-rate of 20 %, power (1-beta) of 0.90 and an alpha of 0.05. The sample size is calculated at patient level, because the primary outcome measure of this study is back beliefs of patients.

### Randomisation

HCPs will be assigned to one of the four clusters. The clusters sequentially receive the intervention, and the moment of intervention rollout within one cluster the unit of randomisation is (Fig. 1). Randomisation is performed by means of computer-generated allocation. Patients are blinded to group assignment.

The allocation is not concealed from HCPs or for the researchers, because the start of the intervention (i.e., organising or receiving the CME training) is an obvious point in time that does not lend itself for blinding. An independent research assistant will perform the allocation sequence, enrolling of participants, and assignment of participants to groups.

### Statistical methods

Data on outcome measures will be gathered at baseline, at 3, 6, and 12 months follow-up. Data will be compared between intervention and control group. Two (longitudinal) analyses will be performed: 1) a crude analysis of outcome measures, and 2) an analysis of outcome measures adjusted for prognostic dissimilarities, confounding, and effect modification. Multilevel analyses will be performed to take into account repeated measurements in participants as well as the effects of clustering in this study. Clusters will be taken into account in this analysis as fixed effects. All analyses will be performed according to the intention-to-treat principle. Multilevel analyses will be performed using MlWin 2.0. Linear and logistic regression analyses will be performed using SPSS.

#### Economic evaluation

An economic evaluation will be performed to measure and analyse the cost differences between the current implementation strategy and passive dissemination of the guideline in relation to the effect differences between the two groups. Direct costs related to health care utility as well as indirect costs related to productivity losses due to absenteeism and presenteeism will be analysed. Costs of the implementation strategy will be measured, valued and analysed using a bottom-up approach. Health care consumption and productivity losses will be valued using Dutch guideline prices.

Cost-effectiveness and cost-utility analyses will be performed from a societal perspective. Functional status and quality of life of the patients will be taken as outcome measures for the economic evaluation. To calculate incremental cost-effectiveness ratios (ICER) and cost-utility ratios, the differences in mean costs between the two groups will be divided by their differences in outcome measures. Confidence intervals (95 %) will be estimated using bootstrapping methods with a minimum of 5.000 replications [23]. Uncertainty of the ICERs will be graphically presented in a cost-effectiveness plane (CE plane), as well as cost-effectiveness acceptability curves (CEAC) [24, 25].

At last, a budget impact analysis will extrapolate the results of the economic evaluation to a period of 5 years in order to estimate the financial costs of applying this implementation strategy on a larger scale. This analysis will be performed from several perspectives (i.e., societal, government, and insurance) and several scenarios for application of this implementation strategy.

## Discussion

This paper presents the design of a stepped-wedge cluster-randomised controlled trial to evaluate the (cost-) effectiveness of a multifaceted strategy to implement the Dutch multidisciplinary care guideline for nonspecific LBP. While back pain is a threat to the quality of (working) life of patients, it’s high prevalence and related costs are an enormous burden to society as well.

It is known that non-specific LBP in certain patients (i.e., those with yellow flags) has an important psychological compound, with beliefs about back pain playing an important role in the development of back pain disability and return to work of LBP patients [14, 15, 26–28]. Various studies have shown that these patients may believe that back pain is a serious, long-lasting, and disabling problem, which in turn influences health outcomes of these patients [29, 30]. It is the health care provider’s task to tackle these negative beliefs and improve patients’ self-management, and thus relieving the high health care utility and costs due to LBP.

However, it may be difficult for HCPs to adhere to this advice and several studies have explored factors for non-adherence to guidelines. These factors include patients’ past experiences of back pain, interpretations of their preferences, and the inclination to give in to patients’ preferences for care that are not evidence based (e.g., referrals for diagnostic imaging) [31, 32]. HCPs will therefore need excellent communication skills to convince patients of the most appropriate treatment course. Furthermore, it is of utmost importance that the various HCPs involved in the treatment of patients with LBP are on the same line regarding this treatment course. Indeed, it is shown that knowledge transfer about evidence-based care is not sufficient to guideline adherence; public education and interdisciplinary consensus are most important to implement guidelines in daily practice [33], and multifaceted approaches have been known to be effective in improving professional practice [34, 35].

A major study performed in Australia in 1997–1999 (“Back Pain: Don’t Take It Lying Down”) has shown that providing informative, activating, and reassuring messages about back pain are effective in changing back beliefs an behaviour, in shifting beliefs about staying active and at work, in improving self-management abilities and reducing health care utility, costs, and disability claims and benefits paid [13]. This study achieved its successes by providing mass media messages, targeted at the general population, by means of television, radio, and printed advertisement. However, more recent and similar mass media studies such as the “Working Backs Scotland” (Scotland 2000–2003) and “Active Back” (Norway 2002–2005) have failed to reproduce results other than little changes in belief change and disability. These studies were based on models that assumed that changing beliefs would change behaviour. However, they did not account for factors that intervene between beliefs and behaviour. A systematic review performed by Mustard and Bielecky in 2007 [36] showed that education via mass media is not enough to illicit health behaviour change, and that a mix of intervention strategies are necessary in order to reach effects similar to those produced by the Australian mass media campaign.

The current study applies the effective component of the Australian campaign, i.e., informative video-messages, and combines it with a more extensive patient intervention, as well as an intervention targeted at professionals.

**Fig. 2:**
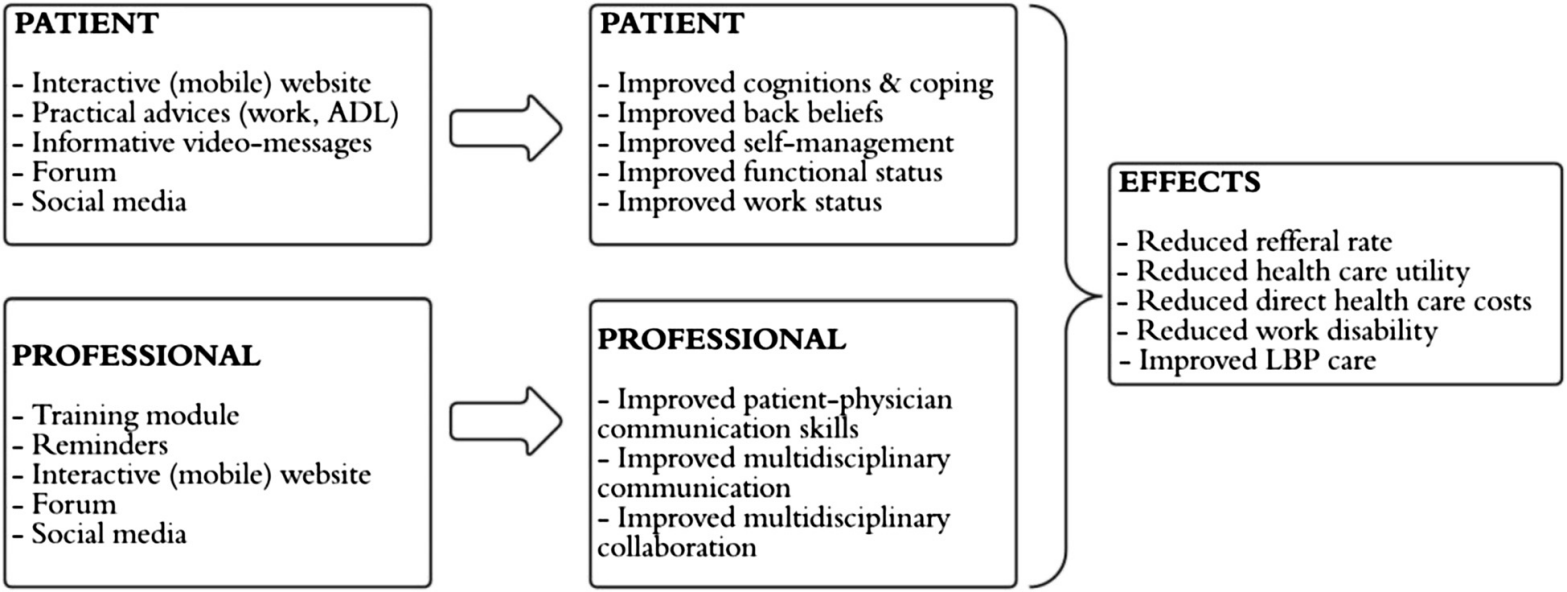
Conceptual model of the multifaceted implementation strategy

The current study tackles well-known factors by applying the knowledge gained from previous back pain campaign, and combining a patient based intervention (aimed at improving back beliefs) with a professional based intervention (a multidisciplinary training module). This combined and innovative strategy is an important strength of the current study, because it applies several intervention components in order to reach various outcomes on patient, professional, and organisational level (Fig. 2). A further strength of the current study is the extensive process evaluation, which will give information that is valuable for interpreting the effectiveness of the results.

Further strengths of the present study include the stepped-wedge cluster randomised design. Because every cluster serves as its own control, but also as control for other clusters, this design allows both between group analyses as well as within-group analyses. Using this design, all clusters will have received the intervention at the end of the study, and thus, participation in this study will be higher. Clustering the participating HCPs allows for prevention of contamination between intervention and control group. Furthermore, this design allows the adaptation and improvement of the intervention strategy based upon experiences gathered during the stepwise implementation process.

Although the stepped-wedge design has shown to be a robust way of evaluating intervention effectiveness in implementation research [37], the current study will likely face some methodological challenges as well. At each point where a new cluster receives the intervention, data collection will be necessary, making this a time-consuming logistical challenge. Furthermore, the analysis of the gathered data will be complex due to these repeated measures in same participants. Inherent to the timing of the intervention rollout being the unit of randomisation, it will be impossible to blind researchers and participating HCPs for sequence allocation.

By combining the results of the intervention effects with the results of the process evaluation, this study will provide insight into the (cost-) effectiveness of the multifaceted implementation strategy as well as insight into patient and HCP experiences with this strategy. The subsequent qualitative subgroup analysis will give insight into the experiences of patients of various ethnic backgrounds with the current implementation strategy. The valuable information gained with this study may prove useful for policy-makers, health care providers, and other researchers who are in the process of reducing the burden of back pain on individuals and societies.
